# Technology-assisted education in graduate medical education: a review of the literature

**DOI:** 10.1186/1865-1380-4-51

**Published:** 2011-08-08

**Authors:** Sharhabeel Jwayyed, Kirk A Stiffler, Scott T Wilber, Alison Southern, John Weigand, Rudd Bare, Lowell W Gerson

**Affiliations:** 1 Department of Emergency Medicine, Summa Akron City Hospital, Akron, OH, USA; 2 Northeastern Ohio Medical University, Rootstown, OH, USA

**Keywords:** education, medical, graduate, computer-assisted instruction, Internet or World Wide Web, simulation, virtual reality

## Abstract

Studies on computer-aided instruction and web-based learning have left many questions unanswered about the most effective use of technology-assisted education in graduate medical education.

**Objective:**

We conducted a review of the current medical literature to report the techniques, methods, frequency and effectiveness of technology-assisted education in graduate medical education.

**Methods:**

A structured review of MEDLINE articles dealing with "Computer-Assisted Instruction," "Internet or World Wide Web," "Education" and "Medical" limited to articles published between 2002-2007 in the English language was performed. RESULTS: The two literature searches returned 679 articles; 184 met our inclusion and exclusion criteria. In 87 articles, effectiveness was measured primarily using self-reported results from a survey of subjects. Technology-assisted education was superior to traditional methods in 42 of the 64 direct comparison articles (66%, 95% CI 53-77%). Traditional teaching methods were superior to technology-assisted education in only 3/64 (5%, 95% CI 1-13%). The remaining 19 direct comparison articles showed no difference. A detailed review of the 64 comparative studies (technology-assisted education versus traditional teaching methods) also failed to identify a best method or best uses for technology-assisted education.

**Conclusions:**

Technology-assisted education is used in graduate medical education across a variety of content areas and participant types. Knowledge gain was the predominant outcome measured. The majority of studies that directly compared knowledge gains in technology-assisted education to traditional teaching methods found technology-assisted education equal or superior to traditional teaching methods, though no "best methods" or "best use" was found within those studies. Only three articles were specific to Emergency Medicine, suggesting further research in our specialty is warranted.

## Background

For decades, medical educators have looked for ways to use computer technology to assist education. In the late 1960s, pioneer medical educators began to develop computer systems that laid the foundation for computer-assisted instruction in medical education [1,2]. These early systems consisted of drill and practice questions and later basic true-false or matching questions. As computer technology improved, so did computer-assisted instruction. Over time, rudimentary computer-aided instruction systems were augmented with multimedia laden systems rich with sound, video and animation

The Internet ushered in a new era that allowed for easy distribution of material, easy access by students and central management by administrators [3,4]. Technologies such as simulation and virtual reality were developed that added new dimensions to instruction. Today, computer-assisted instruction, web-based education simulation and now virtual reality are some of the technologies frequently used to support graduate medical education. We refer to these methods as technology-assisted education.

Multiple studies have been performed to evaluate technology-assisted education in medical education. In a 1992 meta-analysis, Cohen et al. found a "medium-sized effect" of computer-assisted instruction on student learning and recommended more research to identify specific features of computer-assisted instruction that lead to improved student performance [5]. In a structured review published in 2002, Chumley-Jones et al. found that web-based learning (WBL) methods can result in student gains but cautioned "...It is a valuable addition to our educational armory, but it does not replace traditional methods....Educators must define WBL's unique educational contribution."[6] In a 2006 structured review, Cook stated that "Research on WBL in medical education has done little to inform practice."[7]

The questions of when, where and how to best use technology-assisted education have not been adequately addressed by the existing literature. As new technologies emerge, new questions continually arise, further complicating matters. Given the cost in time and money associated with the use of many technology-assisted education systems, lack of knowledge on how to best use this technology places educators in a position of dual jeopardy. Valuable resources could be wasted, and potentially more important, ineffective instructional methods could be unintentionally implemented. Emergency Medicine (EM) educators have to navigate these complicated issues when trying to determine the role of technology-assisted education in their curriculum. EM educators, in particular, are hampered by the relative paucity of EM specific studies and must therefore rely on the pool of information present in the general medical education literature. We examined the current technology-assisted education-related medical literature to determine the scope of use of technology-assisted education, whether technology-assisted education improved knowledge when compared with traditional teaching methods, and whether a "Best Method or Best Use" for technology-assisted education could be identified.

Our objectives were to report the techniques, methods and frequency of use of technology-assisted education in graduate medical education, to evaluate the effectiveness of technology-assisted education in improving knowledge compared to traditional and lecture-based teaching methods, and to determine if there was a consensus or general agreement on a "Best Method or Best Use" for technology-assisted education that could be identified.

## Materials and methods

### Design

We performed a structured review of the medical literature on technology-assisted education.

### Search strategies

Two searches were completed using the National Library of Medicine's PubMed database. The first was performed by the lead author and combined the following keywords using the Boolean search term AND: "Computer-Assisted Instruction," "Internet or World Wide Web," "Education" and "Medical." The search was limited to articles published in the last 5 years in the English language. The 5-year time period was chosen to focus on current teaching method technologies. This search was completed on 30 October 2007 and resulted in 271 citations. The second search was completed by the Information Services librarian using the MeSH terms ("Education, Medical" OR "Education, Medical, Undergraduate" OR "Education, Medical, Graduate" OR "Education, Medical, Continuing") AND "Computer-Assisted Instruction." This search was limited to studies published in the past 5 years in the English language and performed on 6 December 2007, resulting in 408 citations.

### Article selection

We included all studies that involved graduate medical education and computer-assisted instruction, web-based education, simulation, virtual reality or other technologies. Evaluative articles were defined as those articles that conducted an evaluation of the education effectiveness of the technology or process. We excluded descriptive articles (defined as those that described a technology or process but did not assess its educational effectiveness), as well as dental, veterinary, podiatry and patient education articles.

### Article review process

Two investigators conducted a primary review of each article to determine if they were evaluative or descriptive. A third author resolved discrepancies.

Articles underwent a secondary review to determine the method of assessment used to determine effectiveness and collection of other data elements. Group one articles were defined as those that conducted no comparison between educational methods and determined effectiveness through survey/subject self-report. Group two articles were defined as those that conducted no comparison between educational methods and determined effectiveness using some type of objective before and after measurement. Group three articles were defined as those in which a comparison between educational methods was done and effectiveness was measured using an objective method such as a pretest and posttest, checklist, computer log or direct observation. Articles were also reviewed for any information that suggested a proven or generally accepted best method was used in the study. During secondary review, if objective assessment methods were not present, articles were considered descriptive and excluded.

### Data collection

A data collection sheet was developed and pretested on three faculty members who provided feedback on clarity and general usability. Study investigators were then instructed on how to complete the article reviews using the data collection sheet. The five study investigators who performed the article reviews completed a pilot review using the data collection sheet and eight randomly chosen articles from the study sample. The pilot review provided the opportunity to clarify items on the data sheet and article review methods. Feedback from this pilot review was used to further modify the data collection sheet and article review methods.

All articles meeting inclusion criteria were then reviewed by a study investigator and data elements were recorded for each article. If the reviewing investigator had any questions about a data element, the article was reviewed by a panel of investigators consisting of the lead author and two additional investigators. The coding of the data element in question was resolved by the majority opinion of this panel. Data were entered by a research technician into a Microsoft Access database.

### Data analysis

Data were analyzed using Stata^®^, version 11. Data are presented using descriptive statistics (means and proportions) with associated 95% confidence intervals (CI).

## Results

**Figure 1:**
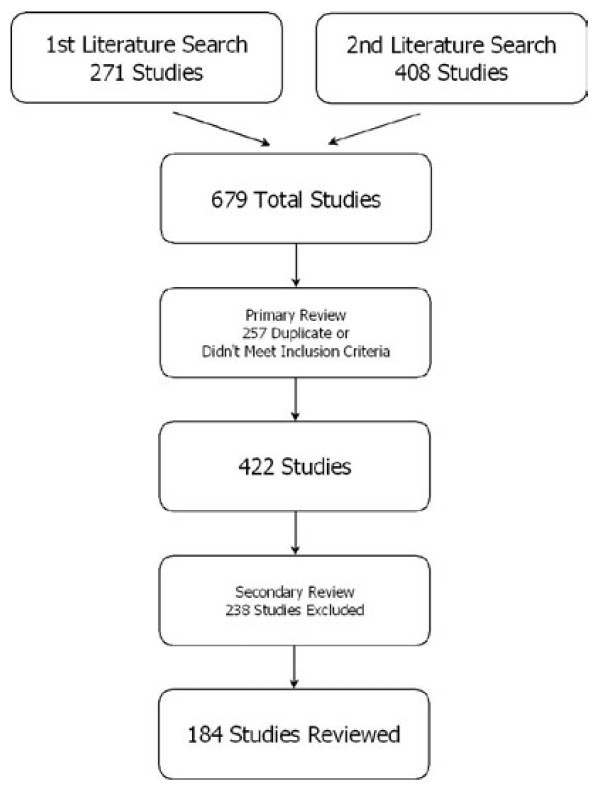
**Article selection and review process**.

The results of the searches and initial review for eligibility are shown in Figure 1. From the 679 studies originally identified in the searches, 257 articles were excluded because of duplication or failure to meet study criteria in the primary review process. During the secondary review, 238 articles were excluded because of nonobjective assessment methods or not meeting inclusion criteria. A total of 184 studies met the inclusion criteria and were reviewed by an investigator. Descriptive data from these studies are shown in Table 1. Of these, 87 articles were group 1 where no comparison was done between educational methods and effectiveness was measured primarily using self-reported results from a survey of subjects; 18 articles were group 2 where no comparison was done between educational methods and objective before and after methods where used to assess effectiveness. There were 79 articles in group 3 (43%, 95% CI 36-50), which consisted of studies in which a comparison was conducted between educational methods and objective methods were used to measure effectiveness (Table 2). Assessment methods commonly used included subject self-assessment by survey, computer log, a pretest and posttest, checklists and direct observation.

**Table 1:** Descriptive study data

	Proportions	% (95% CI)
Category		
Basic science	45/184	24, 18-31
Clinical medicine	125/184	68, 61-75
CME	6/184	3, 1-7
Other	8/184	4, 2-8
Main outcomes studies assessed		
(Some studies assessed multiple outcomes)		
Access	38/184	21, 15-27
Attitude	47/184	26, 19-32
Clinical skills	57/184	31, 24-38
Knowledge	90/184	49, 41-56
Satisfaction	82/184	45, 37-52
Subjects		
Attendings	21/184	11, 7 -17
Medical students	111/184	60, 53-67
Nursing	6/184	3, 1-7
Residents	39/184	21, 15-28
Combination of subjects	7/184	4, 2-8
Descriptive study data		
Resident specialty		
Emergency medicine	3/39	8, 2-21
Internal medicine	5/39	13, 4-27
General surgery	10/39	26,13-42
Pediatrics	6/39	15, 6-31
Radiology	5/39	13, 4-27
Other	3/39	8, 2-21
Combination of residents	7/39	18, 8-34
Technology used		
(Some studies used multiple technologies)	59/184	32, 25-39
	26/184	14, 9-20
CD-ROM/computer based	16/184	9, 5-14
Simulation	94/184	51, 44-59
Virtual reality		
Web based		
Country of origin		
Canada	12/184	6, 3-11
Germany	18/184	10, 6-15
Great Britain	14/184	8, 4-12
United States	105/184	57, 50-64
Others	35/184	16, 11-22

**Table 2:** Description of groups

Group	Number/Group	Primary method of assessment	Comparison with traditional methods
One	87	Survey/self-reported results	None
Two	18	Before/after measurement	None
Three	79	Objective assessment (pretest/posttest, checklist, etc.)	Yes

In 64 of the group three articles (64/184, 35%, 95% CI 28-42), there was a direct comparison between technology-assisted education and traditional teaching methods (Table 3). In the majority of these 64 articles, the subjects were medical students and content area was clinical medicine. Technology-assisted education was superior to traditional teaching methods in 42 of the 64 direct comparison articles (66%, 95% CI 53-77%), traditional teaching methods were superior to technology-assisted education in only 3 of the 64 articles (5%, 95% CI 1-13%), and the remaining 19 showed no difference. No consistent best methods or best uses were identified after review of the articles. A detailed review of the 64 comparative studies (technology-assisted education versus traditional teaching methods also failed to identify a best method or best uses for technology-assisted education.

**Table 3:** 64 comparative articles

Author	Category	Outcomes measured ^A^	Methods compared	Study characteristics B	Number of subjects in study C	Study subjects	Type of resident C	Magnitude of benefit % D	Type of assessment E	Preferred method	Country of study
Uranus et al.	Clinical medicine	Cs,Ap	VR to Sim	Cs	62	Attendings/med students	NR	"Sig Better"	DO	Technology-VR	Austria
Wehrs	Clinical medicine	K,At,S,Ap	Trad to CAI	C,L	38	Attendings	NR	22	T,S	Technology	Germany
Casebeer	CME	K,S,Ap,Ac	Trad to WBL	C,R	210	Attendings	NR	15	T,S	Technology	USA
Short et al.	CME	K	Trad to CAI	C,R	52	Attendings	NR	NRA	S	Standard	USA
Butzlaff et al.	Clinical medicine	K,Ap,Ac	Trad to WBL	C,R	72	Attendings	NR	NA	T,S	No difference	Germany
Forester et al.	Basic science	K	Trad to WBL	C	477	Med students	NR	9	T,S	Technology	USA
Krippendorf	Basic science	K,S,Ap	Trad to CAI/VR	C,Cs	206	Med students	NR	2	T,S	Technology	USA
Hudson et al.	Basic science	K	Trad to CAI	C,R	100	Med students	NR	11	T,S	Technology	UK
Taradi et al.	Basic science	K,At,S,Ap,Ac	Trad to WBL	Cs	220	Med students	NR	10	T,S	Technology	Croatia
Thatcher	Basic science	K,At,S,Ap	Trad to CAI	Cs	22	Med students	NR	22	T,S	Technology	USA
McNulty	Basic science	K	Trad to CAI	Cs	130	Med students	NR	2	T,S, CL	Technology	USA
Noimark et al.	Clinical medicine	K	Trad to WBL	Cs	346	Med students	NR	15	T	Technology	UK
Leong et al.	Clinical medicine	K,S	Trad to CAI	Cs	379	Med students	NR	12	T,S	Technology	USA
Prinz	Clinical medicine	K	Trad to CAI	C,R	172	Med students	NR	7/16/19*	T,S	Technology	Austria
Degnan et al.	Clinical medicine	K	Trad to CAI	C,R	48	Med students	NR	NRA	DO,CL	Technology	UK
Burgess et al.	Clinical medicine	K,At,S,Ap,Ac	Trad to WBL	Cs	91	Med students	NR	NRA	S	Technology	UK
Vivekananda-Schmidt et al.	Clinical medicine	K,Cs,At,S,Ap	Trad to CAI	C,R,Cs	354	Med students	NR	"Sig better"	DO, S	Technology	UK
Callas et al.	Clinical medicine	S,Ap,Ac	Trad to WBL	Cs	903	Med students	NR	NRA	S	Technology	USA
Ganai et al.	Clinical Medicine	Cs	VR to Trad	C,R	19	Med students	NR	40	DO, T	Technology	USA
Duque et al.	Clinical medicine	Cs	Trad to WBL	C,L	100	Med students	NR	NRA	T,S	Technology	Canada
Stolz et al.	Clinical medicine	K,S,Ap,Ac	Trad to WBL	-	NR	Med students	NR	10	T,S	Technology	Germany
Duque et al.	Clinical medicine	Cs,At,S	Trad to WBL	C,R	133	Med students	NR	14	T,S,CKL	Technology	Canada
Vash et al.	Clinical medicine	K	VR to TRAD	C,R	48	Med students	NR	NRA	T	Technology (in 1/4 content areas)	Iran
Schilling et al.	Clinical medicine	K,S,Ap	Trad to WBL	C,R	238	Med students	NR	26/46*	T,S	Technology	USA
Roesch et al.	Clinical medicine	K,S	Trad to WBL	L	3050	Med students	NR	10	T,S	Technology	Germany
Ridgway et al.	Clinical medicine	K,S,Ap,Ac,At	Trad to WBL	Cs	88	Med students	NR	4/10*	T,S, CL	Technology	Ireland
Qayumi et al.	Clinical medicine	K,S,Cs,At,Ap,	Trad to CAI	C,R	99	Med students	NR	Sig improvement	T,S,DO	Technology	Japan
Shokar et al.	Clinical medicine	K,Cs	Trad to WBL	C	179	Med students	NR	5/4*	T,DO	Technology	USA
Glittenberg et al.	Clinical medicine	K,At,S	Trad to CAI	C,R	140	Med students	NR	17	T,S	Technology	Austria
Friedl et al.	Clinical medicine	K,Cs	Trad to CAI	C	195	Med students	Surg	15/18 Cs only	T,DO	Standard (no diff K, CAI > Cs)	Germany
Engum et al.	Clinical medicine	K,Cs,S	Trad to VR	C,R	163	Med students	NR	NRA	T,S, CKL	Standard	USA
Hariri et al.	Basic science	K,S	Sim to Trad	C,R	29	Med students	NR	NA	T,S	No difference	USA
Kumar et al.	Basic science	K,At,S,Ap,Ac	Trad to CAI	Cs	212	Med students	NR	NRA	S	Technology	Australia
Cox et al.	Clinical medicine	K,At,S	Trad to WBL	C,R	100	Med students	NR	NA	T,S	No difference	USA
Chou et al.	Clinical medicine	K	VR to Trad	C,R	16	Med students	NR	NA	CKL	No difference	USA
Hahne et al.	Clinical medicine	K,At,S	Trad to CAI	C,R	167	Med students	NR	NA	T,S	No difference	Germany
Curran et al.	Clinical medicine	K,Cs,At,S	Sim to Trad	C,R	60	Med students	NR	NA	T,S, CKL	No difference	Canada
Wahlgren et al.	Clinical medicine	K,At,S,Ap,Ac	Trad to CAI	C,R,L	116	Med students	NR	NA	T,S, CL	No difference	Sweden
Raij et al.	Clinical medicine	Cs,Ap	VR to Trad	C	82	Med students	NR	NA	S,CL	No difference	USA
Nackman et al.	Clinical medicine	Cs	Trad to WBL	Cs	198	Med students	NR	NA	T	No difference	USA
Karnath et al.	Clinical medicine	Cs	Trad to CAI	C,R	192	Med students	NR	NA	T	No difference	USA
Feeg et al.	Clinical medicine	K,At	Trad to CAI	C,R	125	Nurses	NR	10	T	Technology	USA
Anderson et al.	Clinical medicine	K,Cs	Trad to WBL	Cs	180	Nurses	NR	NA	S	No difference	USA
Dee et al.	Basic science	K	Trad to CAI	L	98	Other	NR	NRA	S	Technology	USA
Errichetti et al.	Clinical medicine	Cs	Trad to CAI	C,R	40	Med students	NR	NRA	CL	Technology	USA
Ryan et al.	Basic science	K	Trad to CAI	C	23	Med students	NR	NA	S	No difference	Ireland
Gold et al.	Basic science	K,S	Trad to CAI	C,R	138	Residents	Surg	NRA	T,S,CL	Technology	USA
Roche et al.	Basic science	K	Trad to CAI	C,R	38	Residents	Peds	11	T,S	Technology	USA
Gold et al.	Basic ccience	K,S	Trad to CAI	C,R	138	Residents	Surg	NRA	T,S,CL	Technology	USA
Barsuck et al.	Clinical medicine	K,Cs	Sim to Trad	Cs	72	Residents	NR	47/31*	T,CL	Technology	Israel
Xiao et al.	Clinical medicine	K,Cs	Trad to CAI/WBL	C,R,Cs	50	Residents	EM/Surg	41	CL	Technology	USA
Knoll et al.	Clinical medicine	Cs	Sim to Trad	C,R	30	Residents/attendings	GU	NRA	CL,CKL	Technology	Germany
Mahnke et al.	Clinical medicine	Cs	Trad to CAI	Cs	40	Residents	Peds	21	T	Technology	USA
Park et al.	Clinical medicine	Cs	Sim to Trad	C,R	24	Residents	IM/Surg	NRA	CL,CKL	Technology	Canada
Schijven et al.	Clinical medicine	K,S,Ap	VR to Trad	C	24	Residents	Surg	NRA	DO, S	Technology	Netherlands
Maiss et al.	Clinical medicine	Cs	Sim to Other	C,R	35	Residents	IM	NRA	CL	Technology	France
Jonas et al.	Clinical medicine	Cs	Sim to Trad	C,R	14	Residents/med students	Ophthal	NRA	CKL	Technology	Germany
Sedlack et al.	Clinical medicine	Cs	Sim to Trad	C	38	Residents	IM	NRA	S,CKL	Technology	USA
Corton et al.	Basic science	K,S,Ap	Trad to CAI	C,R,L	39	Residents	GYN	NA	T,S	No difference	USA
Jowett et al.	Clinical medicine	Cs	Trad to CAI	C,R,L	30	Residents	Surg	NA	T	No difference	Canada
Chung et al.	Clinical medicine	K	Trad to WBL	C,R,L	63	Residents	EM	NA	T,S,CL	No difference	USA
Davis et al.	Clinical medicine	K,At	Trad to CAI	C,R	55	Residents	NR residents	NA	T,S	No difference	UK
Ferguson et al.	Clinical medicine	K	Trad to WBL	O	19	Residents	Surg	NA	T,CL	No difference	USA
Bridgemohan et al.	Clinical medicine	K,At,S	Trad to CAI	R	46	Residents	Peds	NA	T,S	No difference	USA

*Legend: outcomes measured A*

**Ac: ***Access (can students get to and use the learning material); ***Ap:***Applicability (can you teach with this method); ***At: ***Attitude; ***Cs: ***Clinical Skills (e.g.: H+P skills, EKG, CXR interpretation); ***Cs: ***Cost; ***K: ***Knowledge retention/learning; ***O: ***Other; ***S: ***Satisfaction*.

*Legend: study characteristics B*

**C: ***Controlled (there was a control group); ***Cs: ***Cross sectional (single point in time); ***L: ***Longitudinal (more than one point in time); ***O: ***Other; ***R: ***Random (randomization of groups.)*

*Legend: number of subjects in study/type of resident C*

**NR: ***Not reported*

*Legend: magnitude of benefit % D*

**NRA: ***Not readily available; ***NA: **Not applicable; *Multiple measurements

*Legend: type of assessment E*

**DO: ***Direct observation; ***T: ***Test; ***S: ***Survey; ***CL: ***Computer log; ***CKL: ***Check list*.

Most articles evaluated technology-assisted education with regard to clinical medicine (123/184, 67%, 95% CI 59-74%) and basic science education (44/184, 24%, 95% CI 18-31%). Knowledge gains were the most common outcome assessed by the literature (90/184, 49%, 95% CI 42-56%). Other outcomes commonly assessed included satisfaction (82/184, 45%, 95% CI 37-52%), clinical skills (57/184, 31%, 95% CI 24-38%), attitudes (47/184, 26% 95% CI 19-32%) and access to technology-assisted education (38/184, 21%, 95% CI 15-27%). The participants of the studies were predominantly medical students (111/184, 60%, 95% CI 53-67%) and resident physicians (39/184, 21%, 95% CI 15-28%). Of the resident based studies, there was no predominant specialty, with only three studies (0.02%, 95% CI 0.003-0.05%) specific to Emergency Medicine.

## Discussion

Technology-assisted education is used in graduated medical education across a variety of content areas and subject types. Content areas ranged from basic science subjects such anatomy and pathology, to clinical medicine (training in procedures, diagnosis and management), and even to cognitive skills and attitudes [8-13].

Computer and Internet-based methods were the most commonly used modalities followed by simulation and virtual reality. Clinical studies were the most common type of study. The most common study subjects were medical students followed by residents and attending physicians. Only three articles were related to the specialty of emergency medicine. The majority of articles were from authors based in the US and attempted to measure gains in knowledge or skills. Many studies sought to measure satisfaction and attitudes toward the main intervention. The majority of studies that directly compared traditional teaching methods to technology-assisted education found technology-assisted education equal or superior to traditional teaching methods. We did not find any particular method or use of technology-assisted education that could be described as a "Best Method." Assessment methods commonly used included subject self-assessment by survey, computer log, a pretest and posttest, and direct observation.

Technology-assisted education has the potential to enrich learning in ways not possible using traditional methods of instruction [14]. Technology-assisted education allows individualized self-paced learning, improved assessment, evaluation and feedback while increasing learner's exposure to other instructional material [2,15,16]. Additionally, technology-assisted education provides for inherent efficiency in the administration of educational material that encompasses development, distribution, retrial, storage and communication. The desire to harness these advantages and the other useful features of technology-assisted education is a driving force behind the efforts of medical educators to determine the most effective use technology-assisted education.

Our study confirms the findings of previous studies that technology-assisted education can result in knowledge improvement [5,6,17]. Eighty-seven (87) articles in our study assessed gains by surveying subjects and asking for their self-assessment of improvement in knowledge or skills after exposure to the study method. This may be an inaccurate technique to determine the effectiveness of the teaching method used in the study. Kirkpatrick describes a four-level approach to evaluate training programs. These levels are: Reaction, Learning, Behavior and Results (See Table 4) [18]. A subject's self-reported sense of improvement is likely a measure of the Reaction level and not a true measure of learning. Student attitude and acceptance of a training method are important precursors to the success of any educational method. However, studies that relied solely on self-assessment to determine the degree of learning may have missed the mark and may be of limited value as a result.

**Table 4:** Kirkpatrick's four levels of evaluation

Level	Focus of level	Possible measurement method
Reaction	Student's perception of or satisfaction with training method	Survey, focus groups
Learning	To measure if students' knowledge/skills/attitude changed	Control group Objective pretest/post test of knowledge/skills Direct observation Checklist
Behavior	Determine if the new knowledge/skills/attitudes are being used by the student	Control group Direct observation checklist Before/after interview or survey of student's direct contacts or supervisors
Results	The trainings impact on the organization	An improvement in quality, productivity, reduction in cost, increase profit or some other tangible benefit to the organization

Adapted from Kirkpatrick [18]

The 64 studies that compared traditional teaching methods with technology-assisted education used objective measurements to determine learning outcomes such as a pretest and posttest, checklist and computer log. Two-thirds found technology-assisted education superior to traditional teaching methods. Why or when technology-assisted education might be better than traditional teaching methods was not always predictable. Visualization has been shown to improve learning [19]. The teaching of subject matter that consists of complex associations or difficult to demonstrate spatial relationships using standard methods can be enhanced with computer-assisted instruction. Some studies we reviewed provide insight on this illusive issue. Thatcher compared the use of computer-assisted instruction to traditional methods to teach medical students about DNA replication and found that the computer-assisted instruction group performed 22% better on the posttest than the traditional textbook group [20]. Thatcher suggested the multimedia teaching that was possible with computer-assisted instruction enriched learning. Computer-assisted instruction allowed the complicated sequence of steps and the spatial relationships associated with DNA replication to be presented in a three-dimensional format, something that was not possible with a two-dimensional textbook.

An article we reviewed by Glittenberg and colleagues describes the development and use of a three-dimensional interactive computer-assisted instruction program designed to teach students the basics of the human oculomotor system [21]. This teaching program included information about the main and auxiliary functions of each extra-ocular eye muscle, which eye muscles are active during any given movement of the eye, the path of the oculomotor cranial nerves, the symptoms of cranial nerve paralysis, as well as symptoms of various neurological pathologies. The authors compared this teaching program to standard teaching methods that used textbooks, pictures and diagrams. Formal assessment methods found that the computer-assisted instruction group performed 20% better than the traditional teaching group. Glittenberg noted that the complex material could be demonstrated in a richer fashion using computer-assisted instruction than was possible with traditional teaching methods and commented that... "These findings suggest that high-quality 3D animations may help students and physicians, especially those with low-spatial abilities, to conceptualize abstract topics in medicine and ophthalmology in a way that makes it easier for them to understand and remember these topics." The conclusions by Thatcher and Glittenberg are supported by Mayer who contends that multimedia learning made possible with technology-assisted education allows information to be presented to the student using multiple sensory pathways [14]. This aids the students' development in understanding the material.

However, improvement in student performance with technology-assisted education was not universal in the studies we reviewed. About a third of studies that compared technology-assisted education with traditional teaching methods found no difference in student performance. Again, it was not always clear why these different teaching methods produced the same results. Corton and colleagues developed an interactive computer-based method to teach pelvic anatomy and compared it to a conventional paper-based teaching method [22]. Study subjects were randomized and pretests, posttests and follow-up tests were used to assess learning. They found no difference in knowledge gains between the technology-assisted education and traditional teaching method group despite that fact that most students preferred the technology-assisted education method. The authors commented that the small number of participants (39) and the fact that many participants had technical difficulty viewing the animations and videos may have impacted the results. In a similar study, Forester examined the effects of four supplemental programs on learning of gross anatomy [23]. The four supplemental programs were student teaching assistance, direct study, weekly instructor review and a web-based anatomy program. There was no significant difference between the interventions as all groups showed improvement in knowledge compared to controls. Given the complex, spatial relationships associated with anatomy, one might have expected the web-based group to outperform the other groups. However, there was no explanation advanced as to why all four groups performed the same. Interestingly, students in this study preferred the method that provided more direct contact with the instructors.

Other investigators in our review, such as Davis et al., who conducted a study on teaching evidence-based medicine, and Cox et al., who studied teaching concepts related to the underserved, found no difference between technology-assisted education and traditional teaching methods, suggest that technology-assisted education methods could serve as a possible alternative to lecture [24,25]. These authors note the potential savings in time related to student/instructor travel and preparation of content as well as the ability to standardize content and teaching methods [24,25]. An additional advantage of the technology-assisted education methods is that these methods can be made available continuously for use when convenient to the students. Whether "no difference" means that instructional methods are interchangeable is an open question that is probably best determined by further study.

We were unable to identify specific information in the articles we reviewed that lead us to a "Best Method or Best Use" for technology-assisted education. We had hoped that the 64 studies that directly compared technology-assisted education to traditional education methods would provide information regarding this question. In many of these reviewed studies, authors offered opinions similar to those advanced by Thatcher and Glittenberg within their papers. Additional light is shed on the issue by other investigators. Cook et al. published an article that reviews ten steps to effective web-based learning [26]. Issenberg and colleagues, in a systemic review of simulation-based education, identified 20 important guidelines they recommend authors should adhere to when conducting research on simulation-based education [27]. The efforts of these authors provide more pieces to the puzzle that help bring the answer to the question of how best to use technology-assisted education into better focus.

When motion pictures were first invented, Thomas Edison is reported to have predicted that motion pictures would revolutionize education. Experts agree it did not [14]. Similar unfulfilled claims were made when other technologies like radio and television were invented [14]. We certainly should not repeat the mistakes of our predecessors. Our study found that technology-assisted education is used across the wide spectrum of graduate medical education underscoring the reality that technology-assisted education is here to stay and will likely change teaching and learning in ways we cannot predict. Consider the experience of Piemme, who in a 1988 article expressed excitement at the potential uses of a new technology called the CD-ROM [1].

Despite a body of research that suggests technology-assisted education can improve knowledge gains and student achievement, there remains difficulty in establishing technology-assisted education's exact role in the current curriculum and the degree to which it can replace traditional teaching methods [5-7,15,28]. Rapidly evolving computer technology presents educators with potential new methods of instruction on a near continuous basis [1,29,30]. This is one factor that makes it difficult to determine the best use of technology-assisted education. An additional confounder may be faulty use of technology-assisted education by educators. We have been constantly reminded that technology-assisted education is a tool that needs to be used properly if it is to be effective. Educators should seek resources that explain how to effectively use technology-assisted education before investing time and money on its application (see Additional readings) [26,27,31-34].

Another barrier to determining technology-assisted education's role in the curriculum is the quality of the published research in this domain. Our study is similar to other studies that showed conflicting results when technology-assisted education methods were compared to traditional methods [6,17]. In the studies we reviewed, there was a wide variety of subjects, settings and assessment methods. Many investigators have voiced concerns about the persistent limitations, often systemic, that exist in studies on computer-based education [5-7,17,35]. Some authors have suggested that studies that compare traditional non-computer teaching methods to computer-based learning are fundamentally flawed because no true comparison exists between the two [36,37]. Friedman and Cook suggest it would be more productive to study one form of technology-assisted education versus another or study how to effectively integrate and measure technology-assisted education's impact on learning [36,37].

We had difficulty identifying the core elements in many of the studies we reviewed (see Limitations). Concordant with this finding, a number of authors have recommended a more organized and programmatic approach to research in the area of technology-assisted education (and medical education in general) to advance our understanding in this domain [36-39]. Detailed information about all aspects of the study as well as specific goals and objectives combined with formal, valid assessment methods are critical precursors for an effective study.

Medical educators are tasked with teaching competencies established by the Accreditation Council of Graduate Medical Education (ACGME). These competencies have been described elsewhere [40]. We would recommend authors align their research with the ACGME core competencies and identify which core competencies are being addressed by the study. Competency can be defined as the specific knowledge, skill and attitude needed to complete a task correctly. Authors should identify which aspect of competency their study deals with (knowledge, skill or attitude) and precisely how the competency is measured. A more organized approach to technology-assisted education research may allow educators across specialties to learn from each other. This could facilitate a more comprehensive cross-specialty understanding of how to best use technology-assisted education. If, for example, researchers in the surgical specialties identified key training elements of technology-assisted education need to master the skills of laparoscopic procedures, these training elements might be important in learning other skills such as endotracheal intubation or lumbar puncture. Similarly, if one specialty identified the key training elements of professionalism that could be taught or measured using technology-assisted education, all specialties would benefit from this knowledge. More structured, programmatic research maybe the best way to foster transfer of training knowledge from one specialty to another and may be the only way to identify a "Best Method or Best Use" for technology-assisted education, something that has eluded medical educators for decades.

## Limitations

Our study has a number of limitations. We attempted to conduct a review of the medical literature covering a 5-year period. The sensitivity of literature searches varies and can be improved when conducted by librarians [41]. Despite using the services of experienced research librarians for our search, some articles may not have been identified and therefore are not included in the body of literature.

The wide variety of study designs, settings, subjects, assessment and reporting methods made combining results impossible. This heterogeneity of outcomes precluded a meta-analysis from being performed [42]. Additionally, reporting methods used in some studies made data abstraction difficult. In some studies, it was difficult to determine core elements such as who the subjects were because of vague descriptions and incomplete definitions. Despite the use of a study panel to resolve disagreements associated with data abstraction, errors could have been made. Additionally, we did not independently evaluate the original author's results as this was beyond the scope of our review.

## Conclusions

Technology-assisted education is used in graduate medical education across a variety of content areas and subject types. Studies in our review show technology-assisted education can result in improvements in knowledge. Sixty-seven percent (67%) of studies that directly compared knowledge gains in technology-assisted education to traditional teaching methods found technology-assisted education equal or superior to traditional teaching methods. Only three articles dealt primarily with EM. This suggests further research in our specialty is warranted. We would recommended EM educators follow programmatic research methods to avoid limitations found in other studies and consider aligning their research with the ACGME core competencies.

## Article Summary Key Questions

### Why is the topic important?

Technology-assisted education is widely used in graduate medical education. Technology-assisted education needs to be used correctly if it is to be effective. Otherwise, valuable training time and resources could be wasted.

### What does this study attempt to show?

Our study attempted to determine the scope of use of technology-assisted education, whether technology-assisted education improved knowledge when compared with traditional teaching methods, and whether a "Best Method or Best Use" for technology-assisted education could be identified.

### What are the key findings?

-Technology-assisted education can improve knowledge. However, use of technology-assisted education does not guarantee knowledge gains as approximately one third of studies did not show improvement in knowledge gains.

-Many articles in our study (87) assessed gains by surveying subjects and asking for their self-assessment of improvement in knowledge or skills after exposure to the study method. This may be an inaccurate technique to determine the effectiveness of the teaching method used.

- Despite years of use and multiple studies, a "Best Method" or "Best Use" of technology-assisted education was not found.

- Only three articles dealt primarily with Emergency Medicine. This suggests further research in our specialty is warranted. We would recommended EM educators follow programmatic research methods to avoid limitations found in other studies and consider aligning their research with the ACGME core competencies.

### How is patient care impacted?

Improvements in instructional methods should result in enhanced competency by physician thereby improving patient care.

## Competing interests

The authors declare that they have no competing interests.

## Authors' contributions

All authors have made substantial contributions to the intellectual content of the paper. SJ, KAS, STW contributed to the conception and design, acquisition of data, analysis and interpretation of data, statistical analysis, drafting of the manuscript, critical revision of the manuscript for important intellectual content. AS, JW, RB contributed the acquisition of data, analysis and interpretation of data, administrative and technical support. LWG contributed to the conception and design, drafting of the manuscript and supervision.
